# A meta-analysis of the greenhouse gas abatement of bioenergy factoring in land use changes

**DOI:** 10.1038/s41598-018-26712-x

**Published:** 2018-06-04

**Authors:** M. El Akkari, O. Réchauchère, A. Bispo, B. Gabrielle, D. Makowski

**Affiliations:** 1 INRA, DEPE, 147 rue de l’université, 75338 Paris Cedex 07, France; 2 ADEME, Direction Productions et Energies Durables - Service Agriculture et Forêt, 20, Avenue du Grésillé BP 90406 49004, Angers Cedex 01, France; 3 INRA, InfoSol, 2163, avenue de la Pomme de Pin, 45075 ORLEANS cedex 2, France; 4 UMR Ecosys, INRA, AgroParisTech, Université Paris-Saclay, 78850 Thiverval-Grignon, France; 5 INRA, UMR 211 Agronomie, INRA, AgroParisTech, Université Paris-Saclay, 78850 Thiverval-Grignon, France

## Abstract

Non-food biomass production is developing rapidly to fuel the bioenergy sector and substitute dwindling fossil resources, which is likely to impact land-use patterns worldwide. Recent publications attempting to factor this effect into the climate mitigation potential of bioenergy chains have come to widely variable conclusions depending on their scope, data sources or methodology. Here, we conducted a first of its kind, systematic review of scientific literature on this topic and derived quantitative trends through a meta-analysis. We showed that second-generation biofuels and bioelectricity have a larger greenhouse gas (GHG) abatement potential than first generation biofuels, and stand the best chances (with a 80 to 90% probability range) of achieving a 50% reduction compared to fossil fuels. Conversely, directly converting forest ecosystems to produce bioenergy feedstock appeared as the worst-case scenario, systematically leading to negative GHG savings. On the other hand, converting grassland appeared to be a better option and entailed a 60% chance of halving GHG emissions compared to fossil energy sources. Since most climate mitigation scenarios assume still larger savings, it is critical to gain better insight into land-use change effects to provide a more realistic estimate of the mitigation potential associated with bioenergy.

## Introduction

The rapid development of first generation biofuels such as ethanol and biodiesel, which use food crops as feedstocks, has become controversial in the last decade because of the unintended consequences of the underlying policies on food prices and land use worldwide. Biofuel expansion creates an additional demand for agricultural commodities which impacts global markets and may interfere with food security^1^. It also increases the overall pressure on agricultural land since the displacement of food crops by bioenergy crops not only leads to direct land use changes in the region where the feedstock is grown but also to indirect land use changes (iLUC) in other parts of the world, to compensate for the foregone production of food commodities^2,3^. Compensation may involve either an intensification of existing cropland, to increase the output of biomass per unit area, or the conversion of pastures, forests and peat land to arable land^4^. These consequences are usually associated with detrimental effects on the environment, such as increased emissions of GHG and biodiversity depletion from the conversion of natural ecosystems^5^. Even though they remain controversial because of the difficulty in tracking their occurrence^6^, indirect LUC effects are likely to reduce the potential benefits of biofuel chains, in particular regarding GHG emissions^7^.

Although review articles on this topic were recently published^8,9^, none of them involved a systematic survey of literature. As a consequence, it is difficult to derive consistent patterns for the effect of LUC on the GHG balance of bioenergy pathways because of differences in the methodology, scope or data sources used in individual studies^10^. Thus, there remains a large uncertainty of the magnitude of these effects, which hampers the policy processes around biofuels and their contribution to both the energy and climate transitions^6,11^.

This study aimed to alleviate the above-mentioned limitations by uncovering consistent trends via a novel, systematic review of scientific literature of LUC (whether direct or indirect) in relation to biomass development. A quantitative, meta-analysis approach was used to capture the relative differences in GHG intensity between the bioenergy chains and their fossil counterfactuals. Thus, the main focus of this study was the potential GHG abatement potential related to the substitution of fossil-based energy sources by their bio-based counterparts. This was quantified through the following effect size *R* in the meta-analysis: $=\frac{Eb-Ef}{Ef}$, where *E*_*f*_ and *E*_*b*_ corresponds to the life-cycle GHG emissions of the fossil and bio-based chains, respectively, expressed per unit of energy output (1 MJ). A negative *R* value implies lower GHG emissions for the bioenergy chain, where a value of −0.5 indicates a 50% reduction compared to the fossil reference. The effect size *R* was successively calculated for each scenario of the 50 articles with two different values of *E*_*f*_ (*i*.*e*., *Ef*_*min*_ and *Ef*_*max*_; see Methods). Mean effect sizes were estimated from a dataset covering 50 articles by fitting mixed-effect models for different groups of scenarios corresponding to different bioenergy end-products (*e*.*g*., electricity or biodiesel), or to different types of LUC (*e*.*g*., conversion of forest to cropland).

## Results

**Figure 1:**
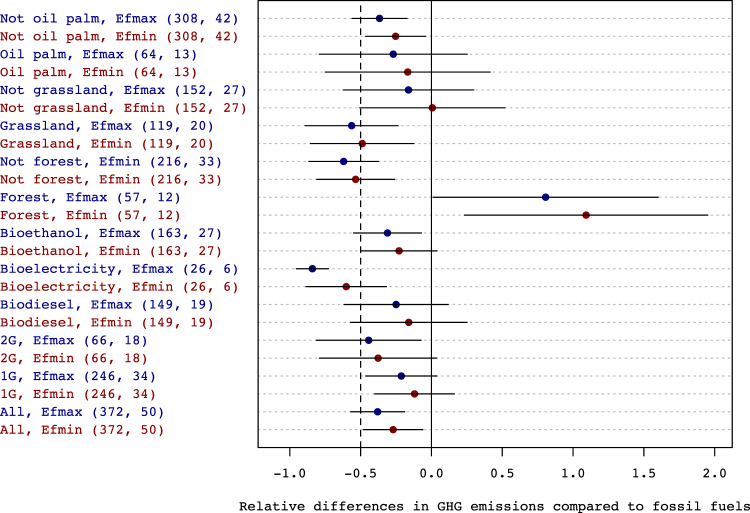
Estimated values of the mean effect-size *R* for different groups of bioenergy scenarios, using the minimum (Efmin) or maximum (Efmax) reference values for the GHG emissions of the fossil counterfactual. The horizontal bars depict 95% confidence intervals. The number of scenarios and the number of articles used in each group are given in brackets. The dotted line corresponds to a 50% GHG reduction level. Key to groups: ‘All’: all scenarios; ‘1G’ and ‘2G’: 1^st^ and 2^nd^ generation biofuels; ‘Forest’: forest as initial land-use; ‘Grassland’: grassland (including degraded pastures) as initial land-use; ‘Palm Oil’: biodiesel from palm oil; ‘Biodiesel’: production of biodiesel; ‘Bioelectricity’: production of bioelectricity; ‘Bioethanol’: production of bioethanol.

The effect size *R* spanned a wide range, encompassing both situations with a very low GHG intensity of biomass compared to fossil fuels, and others with much larger emissions (Table 1). The overall medians stood at −0.59 and −0.65 with the minimum and maximum value of *E*_*f*_, respectively. Thus, bioenergy emitted less GHG than fossil fuels in more than half of the situations assessed. Bioenergy scenarios involving the conversion of forests had the lowest GHG abatement potential, with a median *R* value ranging from 0.57 to 0.84 – which indicates 157 to 184% larger emissions than fossil fuels (Fig. 1). All the other groups had negative *R* values on average, most of which were significantly lower than zero. The effect size was lowest for the “not forest”, “grassland”, and “bioelectricity” groups, followed by “second generation (2G) biofuels”. The estimated average effect size was lower than −0.5 for two groups of land use (“not forest” and “bioelectricity”), regardless of the *E*_*f*_ value, and for three groups of bioenergy end-products (“bioelectricity”, “not forest” and “grassland”) with *Ef*_*min*_. However, *R* was significantly lower than −0.5 only for the “bioelectricity” group with *Ef*_*max*_ (Fig. 1).

**Table 1:** Distribution of the relative differences in GHG intensity between bioenergy and fossil-based equivalents (*R*), calculated with the lower and upper bounds for the emissions of fossil chains (Ef_min_ and Ef_max_).

E_*f*_ Value	R values					
	Min.	1st quartile	Median	Average	3rd quartile	Max.
Minimum bound (Ef_min_)	−4.31	−0.86	−0.59	−0.22	0.14	6.93
Maximum bound (Ef_max_)	−3.96	−0.91	−0.65	−0.34	−0.14	6.09

**Figure 2:**
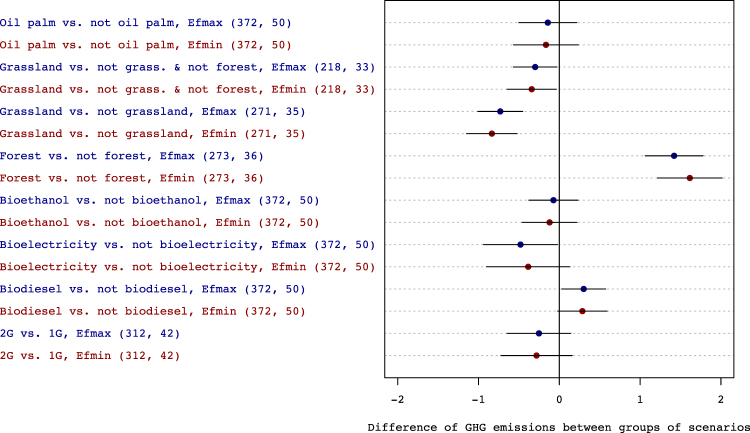
Estimated differences in effect-size *R* between groups of bioenergy scenarios. Either the minimum (Efmin) or maximum (Efmax) reference value for the GHG emissions of the fossil counterfactuals were used. The horizontal bars depict 95% confidence intervals. The number of scenarios and the number of articles used in each group are given in brackets. Key to groups: ‘1G’ and ‘2G’: 1^st^ and 2^nd^ generation biofuels; ‘Forest’: forest as initial land-use; ‘Grassland’: grassland (including degraded pastures) as initial land-use; ‘Oil Palm’: biodiesel from palm oil; ‘Biodiesel’: production of biodiesel; ‘Bioelectricity’: production of bioelectricity; ‘Bioethanol’: production of bioethanol.

Second generation biofuels had higher GHG abatement potentials than their first generation counterparts, especially biodiesel whose potential was 40% lower than the “not biodiesel” group, overall. This difference was statistically significant (*p* < 0.05). The effect size estimated for the “forest” group was more than 100% higher than that estimated for the “not forest” group (Fig. 2). Conversely, the grassland had a significantly lower *R* estimate than the “not grassland” group. Differences between the “grassland” and “not grassland and not forest” groups (i.e., land use scenarios impacting neither grasslands nor forests) were smaller but statistically significant (Fig. 2), regardless of the fossil emissions *E*_*f*_.

**Figure 3:**
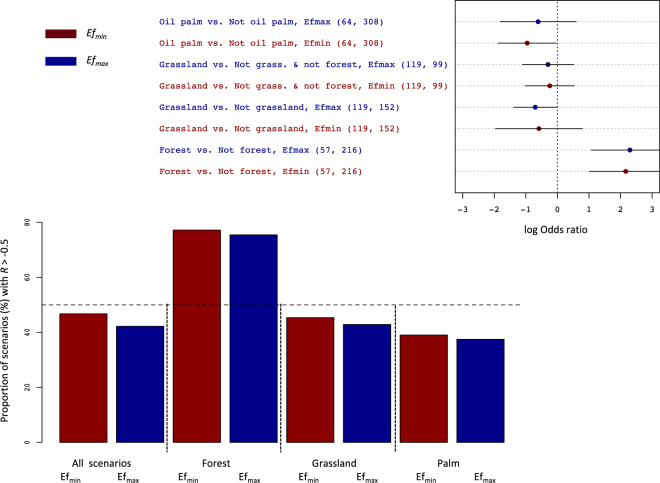
Proportion of scenarios with an effect size value *R* exceeding −0.5 (*ie*, with a GHG abatement under 50%), and estimated differences in the logarithm of the odds ratio between groups of land use scenarios considering both initial and final land uses (top inset). The odds ratio is calculated as the proportion of scenarios with an *R* value greater than −0.5 divided by the proportion of scenarios with a *R* under −0.5. Either the minimum (Efmin) or maximum (Efmax) reference value for the GHG emissions of the fossil counterfactuals were used. The horizontal bars depict 95% confidence intervals. Key to groups: ‘Forest’: forest as initial land use; ‘Grassland’: grassland (including degraded pastures) as initial land use; ‘Palm’: biodiesel made from palm oil.

**Figure 4:**
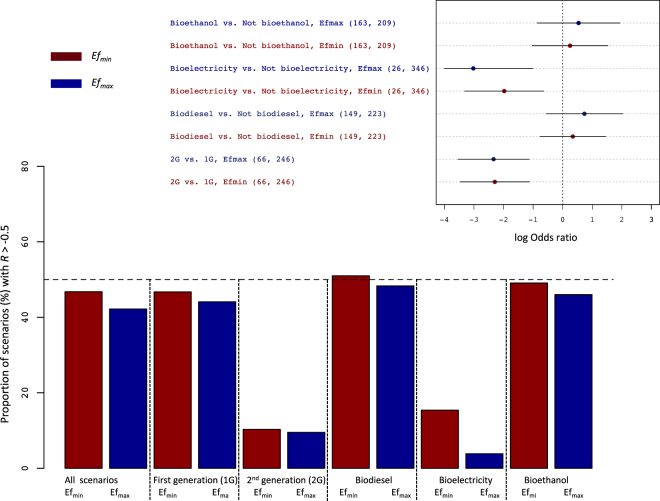
Proportion of scenarios with an effect size value *R* larger than −0.5 (*ie*, with a GHG abatement under 50%), and estimated differences in the logarithm of the odds ratio between groups of scenarios corresponding to different types of bioenergy (top inset). The odds ratio is calculated as the proportion of scenarios with an *R* value greater than −0.5 divided by the proportion of scenarios with an *R* under −0.5. Either the minimum (Efmin) or maximum (Efmax) reference value for the GHG emissions of the fossil counterfactuals were used. The horizontal bars depict 95% confidence intervals.

Except for the groups “forest” and “1G”, most bioenergy scenarios abated GHG emissions by more than 50% (Figs 3, 4). In most of the groups, less than half of the bioenergy scenarios assessed did not achieve a 50% abatement potential relative to fossil fuels (*i*.*e*., had an *R* value exceeding −0.5); (Figs 3, 4). The two exceptions were the “forest” group where this fraction reached 70% (Fig. 3), and the “1G biofuel” group with the minimum value of *E*_*f*_ (Fig. 4).

The logarithm of the odds ratio (ratio of the proportion of scenarios with a *R* > −0.5 to the proportion of scenarios with *R* < −0.5) estimated for the group “Forest vs. not forest” was significantly higher than zero. Thus, bioenergy scenarios not impacting forest ecosystems stood better chances of exceeding a 50% GHG abatement potential than those infringing on forests (Fig. 3). The proportions of scenarios with *R* > −0.5 (i.e., scenarios reducing emissions by less than 50%) were lower in the “2G biofuels” group than in their “1G” counterpart, and in the group “bioelectricity” compared to other types of energy (Fig. 4). The odds ratios indicated that differences were significant for “2G” vs. “1G” biofuels, and for “bioelectricity” vs. “not bioelectricity” (Fig. 4).

## Discussion

Effect sizes were sensitive to the values selected for the emissions of fossil chains (*E*_*f*_), although the latter varied within a narrow range for liquid biofuels (Table 1). Shifting from the minimum value of *E*_*f*_ to its maximum increased the fraction of bioenergy scenarios achieving a 50% GHG abatement from 55% to 62% (Figs 3, 4). For the “1G biofuel” group, this fraction was higher than 50% with Ef_max_ but not with Ef_min_. This has important policy implications since the 50% reduction was made legally-binding in the EU and the USA^12,13^. It emphasizes the need for an accurate assessment of fossil counterfactuals, but also that the probability of certain bioenergy systems complying with these standards is mixed, at best.

Converting forests to biomass crops (whether annual or perennial) appeared as the worst-case scenario (Fig. 3), as could be expected from the differences in carbon pools between the two types of ecosystems, and has been emphasized by previous work^14^. We did not find any significant effect of oil palm on GHG savings (Fig. 3), which is not entirely consistent with primary studies on LUC effects^14,15^. Conversely, the fact that converting grassland was beneficial compared to converting other ecosystems (and in particular cropland and forests – Fig. 3) appears counter-intuitive, given that grasslands are generally deemed carbon-rich^16,17^ compared to cropland. However, some of these grasslands actually corresponded to degraded pastures with lower productivity and thus smaller soil C stocks than the latter land use. In terms of end-products, 2G biofuels and bioelectricity scenarios led to the highest GHG abatements (Figs 1 and 4). This reflects the higher energy-efficiency of these chains compared to liquid fuels^5^.

Although all the studies reviewed used the same system boundaries to account for life-cycle emissions, they resorted to a large variety of methods to assess the consequences of developing bioenergy. Global economic models were only explicitly mentioned in one article (out of 50) while about half of the references did not report a particular method to assess LUC, probably relying on literature data.

Simple methods were also used to account for market mechanisms such as the causal-descriptive approach, which establishes direct correspondences between direct and indirect LUC. Biophysical models^18^ can simulate the biogeochemical processes governing the GHG emissions related to LUC^19^, but were explicitly mentioned in only 10% of the studies. Indirect LUC were ignored in 40% of the bioenergy systems assessed, most of the time on the ground that biomass crops were grown on formerly unproductive land (eg, natural ecosystems or abandoned farmland). Although this ‘no iLUC’ assumption may be debated^15^, it did not significantly affect the effect size *R*: the estimated effect of iLUC inclusion was equal to – 0.12, its standard error was equal to 0.2 and the *p* value higher than 0.5. Separating between direct and indirect LUC effects proved impossible from the information presented in the articles, stemming from the fact that some methods (eg economic modelling) only provide a lumped result in terms of LUC^14^.

These differences in methodologies account for some of the uncertainty around the estimates of GHG abatement for a given bioenergy chain, in addition to local specificities and the types of LUC involved. For instance, economic models tend to result in larger GHG emissions from LUC than the other approaches^18^, and their results are sensitive to modelling assumptions and parameter settings^20^. Inter-comparison of models appears a first step to harmonize methods to assess iLUC and compare bioenergy chains on a similar basis^21^. A key hypothesis also lies in the time horizon during which the C losses incurred upon the conversion of ecosystems to agriculture are amortized. It typically ranges from 20 to 30 years, and has a straightforward effect on LUC-related emissions^22^. This information could be retrieved from all articles but one, with a wide overall range (from 2 to 119 years). Fitting a model relating our response variable *R* to the time horizon (expressed in numbers of years) did not reveal any significant effect of this variable (*p* value > 0.3). Its variations across studies may still explain the larger than average confidence intervals observed in the “forest” group, involving C-rich ecosystems. Another factor likely to affect life-cycle GHG accounting is the procedure used to deal with co-products, which is especially critical for 1G biofuels^10^. The substitution or system expansion method was predominant, with a 40% share, followed by allocation based on energy content (35%) or economic value (17%). Allocation methods had no significant effect on *R* values (p > 0.1; Supplementary Fig. 4).

Although the LUC literature is very recent and its methodologies evolve at a fast pace, no effect of the publication year on the outcomes of the studies could be evidenced (Supplementary Fig. 3). Thus, reducing the uncertainty on the climate benefits of non-food biomass development probably calls for methodological improvements and some degree of standardization, and a more widespread use of state-of-the-art modelling frameworks. This would enhance our understanding of the causal chain from biomass expansion to its impacts on climate, which is paramount to minimizing emissions from LUC and designing efficient bioenergy chains. Land-use models often attribute a larger than 50% GHG abatement potential for bioenergy, with numbers in the 70–90% range for second-generation biofuels or bioelectricity^19,23^. Integrated Assessment Models, which are used to derive socio-economic pathways and emission trajectories to meet given climate targets often even consider bioenergy carbon neutral altogether^24^ – translating as an effect-size of −1.0 here. Although these models may factor in indirect LUC effects, their assessment of bio-based strategies are not aligned with the outcomes of this meta-analysis and should be accordingly revisited. Accounting for LUC effects remains an exercise fraught with uncertainties, as revealed by the large confidence intervals in this meta-analysis, but deserves further improvements to provide an honest view of the mitigation potential of the bio-economy, in the wider perspective of the overall costs and benefits it may incur.

Pending further insights, some patterns already emerge clearly from our comprehensive and quantitative survey. First generation biofuels have the lowest GHG abatement potential of all bioenergy pathways, and stand only about 50% chance of achieving a 50% GHG reduction target. Second-generation biofuels and bioelectricity have a larger greenhouse gas (GHG) abatement potential. Bioenergy value-chains that would impact hitherto unmanaged forest ecosystems should be avoided to maximize the abatement potentials. These results have direct policy implications, whether in terms of the type of pathways that should be supported or the constraints that should be put in place to ensure minimal direct or indirect impact on natural ecosystems.

## Methods

### Literature search and data extraction

A systematic literature search was conducted in the Web of Science using the search equation given in Supplementary Table 1, and led to the identification of 5730 references (published up to Feb. 4^th^, 2015). A subset of 614 articles dealing with non-food biomass production was extracted by screening the titles and abstracts of these references. The 614 articles were scrutinized and 127 of them focusing on the greenhouse gas emissions of bioenergy were selected based on their content. Among this subset, 50 articles were found to report GHG emission data for one or several land use change scenarios characterized by different direct/indirect land use changes and/or by different types of bioenergy chains. A constraint was that articles should cover all the steps of the production to impact chain, and that they include a quantitative evaluation of the GHG emissions of bioenergy (whether for heat production, electricity generation or transport) while fully factoring in LUC effects. Some articles included an assessment of a reference fossil-based energy chain providing the same service as the bioenergy chain at hand. All references relied on the principles of life-cycle assessment encompassing all the stages of the energy systems studied, using a cradle-to-grave approach^11^. The total number of scenarios amounted to 380; 114 of them involved Europe, 110 Southern America, 67 Northern America, 74 Asia, 2 Australia, 1 Southern Africa, and 12 took place in multiple regions. To compare the GHG emissions of fossil and bio-based energy supply the following effect-size *R* was used: $R=\frac{Eb-Ef}{Ef}$, where *E*_*f*_ and *E*_*b*_ corresponds to the life-cycle GHG emissions of the fossil- and bio-based chains, respectively, expressed per unit of energy output (1 MJ). Thus, *R* corresponds to the relative difference in GHG intensity between the bioenergy chain and its fossil counterfactual, and is unitless. To account for the variations of *E*_*f*_ across studies, two values were used for this parameter (*Ef*_*min*_ and *Ef*_*max*_, respectively) corresponding to the lower and upper bounds of the range found in global assessments of fossil chains. Values for *Ef*_*min*_ and *Ef*_*max*_ were 84 and 94 gCO2 MJ^−1^ for biofuels, and 200 and 500 kWh for bioelectricity, respectively^5^, and correspond to the references used for regulation purposes in the European Union and the USA.

### Data analysis and modelling

The two series of effect sizes *R* obtained with *Ef*_*min*_ and *Ef*_*max*_ were analyzed for several groups of scenarios corresponding to different types of bioenergy and land use changes. The following groups were defined: ‘All’: all scenarios; ‘1G’ and ‘2G’: 1^st^ and 2^nd^ generation biofuels; ‘Forest’: forest as initial land-use; ‘Grassland’: grassland (including degraded pastures) as initial land-use; ‘Palm Oil’: biodiesel from palm oil; ‘Biodiesel’: production of biodiesel; ‘Bioelectricity’: production of bioelectricity; ‘Bioethanol’: production of bioethanol. The proportion of *R* values higher than −0.5 and the mean effect size were estimated over all scenarios and for each group of scenarios separately. The significance of the differences among groups was tested using linear mixed effect models and binomial logit mixed models (glmm), including random article effects^25^. The relationship between the year of publication and the effect size was not significant (p = 0.091 and 0.095 with *Ef*_*min*_ and *Ef*_*max*_, respectively (Supplementary Fig. 3). The mixed models were fitted by restricted maximum likelihood^26^with the packages nlme^27^ and lme4 of the R software^28^ (v. 3.2.3). In order to check the robustness of the results to the model assumptions, all mean effect sizes were estimated a second time with a non-parametric method based on 500 bootstrap replicates (articles were used as blocks). The results obtained with the mixed models and with the bootstrap replicates were similar (Supplementary Figs 1, 2).

### Data and code availability

The complete list of references used and the dataset are given in the corresponding sections of the Supplementary Material. The routines used to calculate and analyse the effect sizes are available upon request from the authors.

## Electronic supplementary material

datasert

supplementary materials
