# Supplementary material for: A meta-analysis of the greenhouse gas abatement of bioenergy factoring in land use changes
Source: Sci Rep. 2018 Jun 4;8:8563. doi: 10.1038/s41598-018-26712-x (PMC5986812; doi:10.1038/s41598-018-26712-x)
Supplement: Supplementary file 2 — supplementary materials [file 41598_2018_26712_MOESM2_ESM.pdf]

A meta-analysis of the greenhouse gas abatement of bioenergy factoring in land-use changes

El Akkari M. [1,3,*], Réchauchère O. [1], Bispo A. [2,3], Gabrielle B.[4], Makowski D. [5]

[1] INRA, DEPE, 147 rue de l'université 75338 Paris Cedex 07, France.

[2] ADEME, Direction Productions et Energies Durables - Service Agriculture et Forêt, 20, Avenue du Grésillé BP 90406 49004 Angers Cedex 01, France.

[3] INRA, InfoSol, 2163, avenue de la Pomme de Pin, 45075 ORLEANS cedex 2, France

[4] UMR Ecosys, INRA, AgroParisTech, Université Paris-Saclay, 78850 Thiverval-Grignon, France.

[5] INRA, UMR 211 Agronomie, INRA, AgroParisTech, Université Paris-Saclay, 78850 Thiverval-Grignon, France

* Correspondence to : monia.el-akkari@inra.fr

Supplementary Material

Figure 1. Mean effect size estimates with 500 bootstrap samples (articles used as blocks) with their 95% confidence intervals.

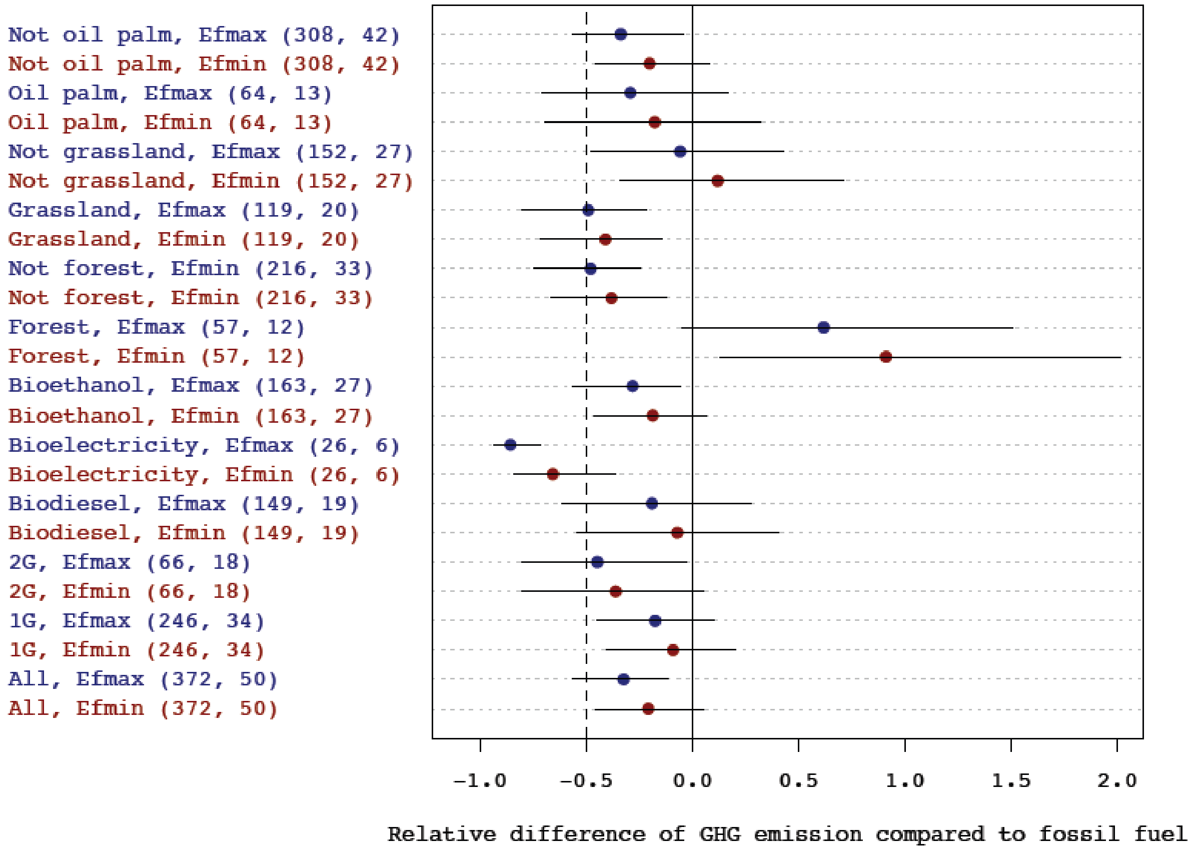

Figure 2. Estimated differences of mean effect sizes between groups of scenarios with 500 bootstrap samples (articles used as blocks) with their 95% confidence intervals

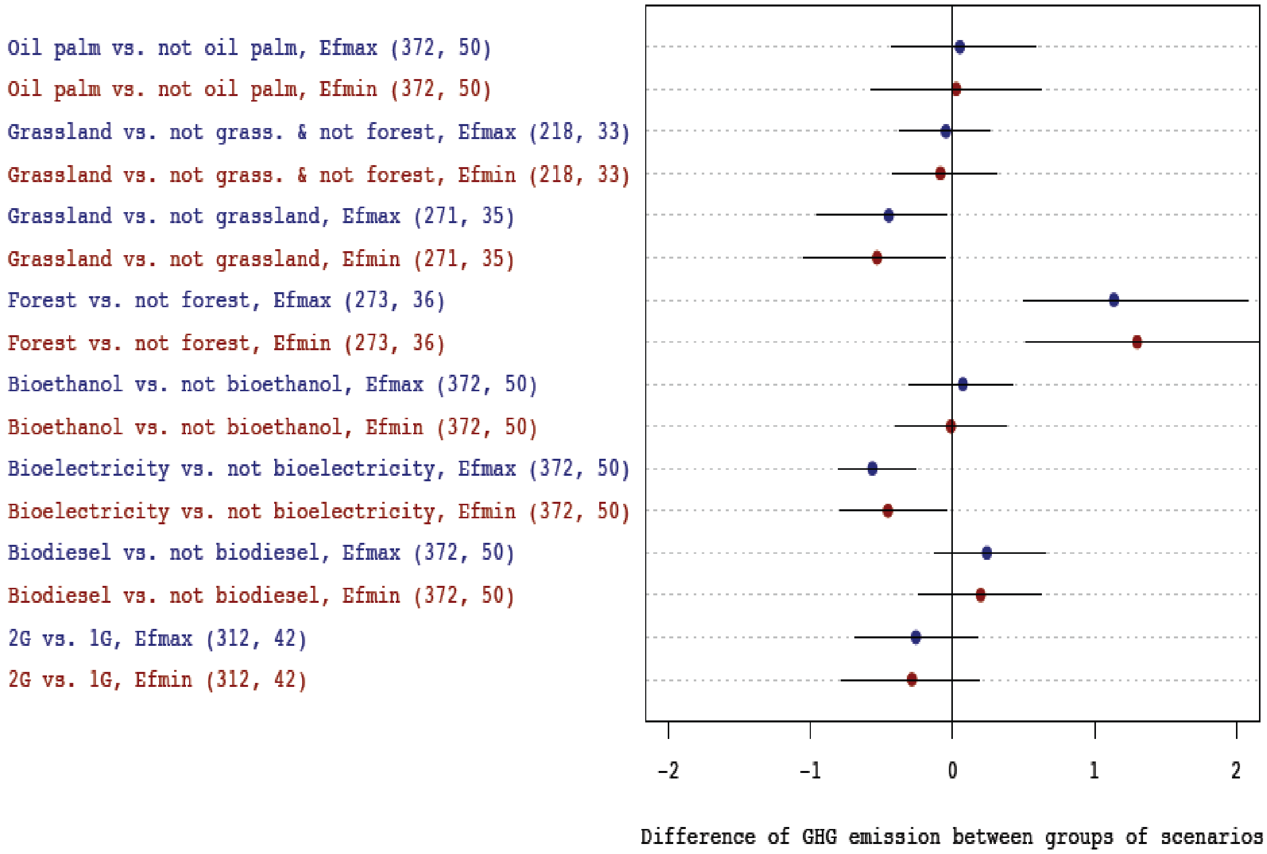

Figure 3. Effect sizes as a function of publication year, using the lower (A) and upper (B) bounds of the fossile GHG emission values (Ef). The blue lines indicate the fitted linear regressions. The *p* values correspond to a test of the absence of year effect on the effect sizes.

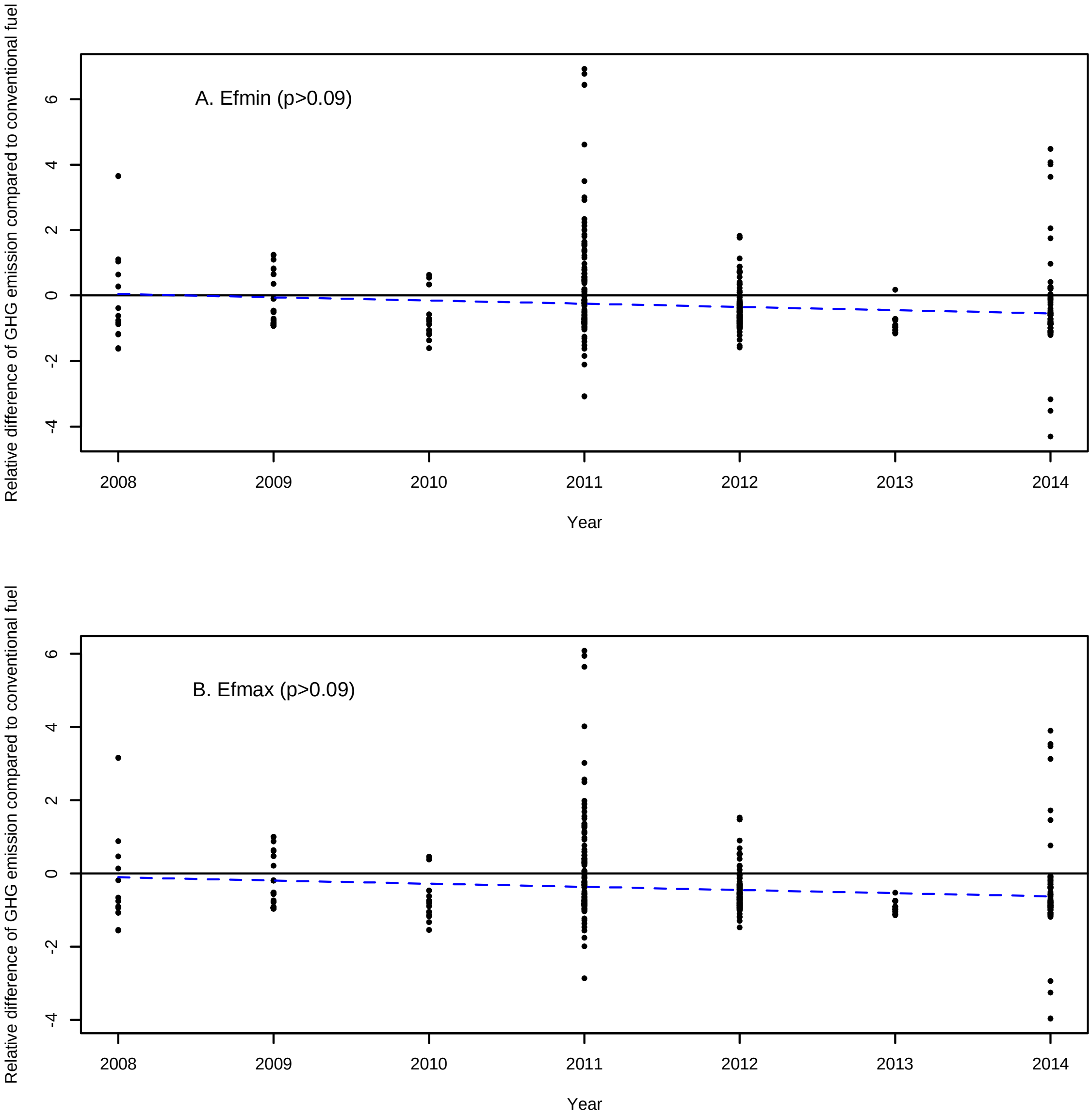

Figure 4. Boxplots of the distribution of the effect sizes as a function of co-product handling method. The figures at the bottom of the graph indicate the number of scenarios involved.

Key to captions : economic, Energy and Mass refer to allocations based on economic value, energy content or mass of the co-products ; S.Expans : System expansion.

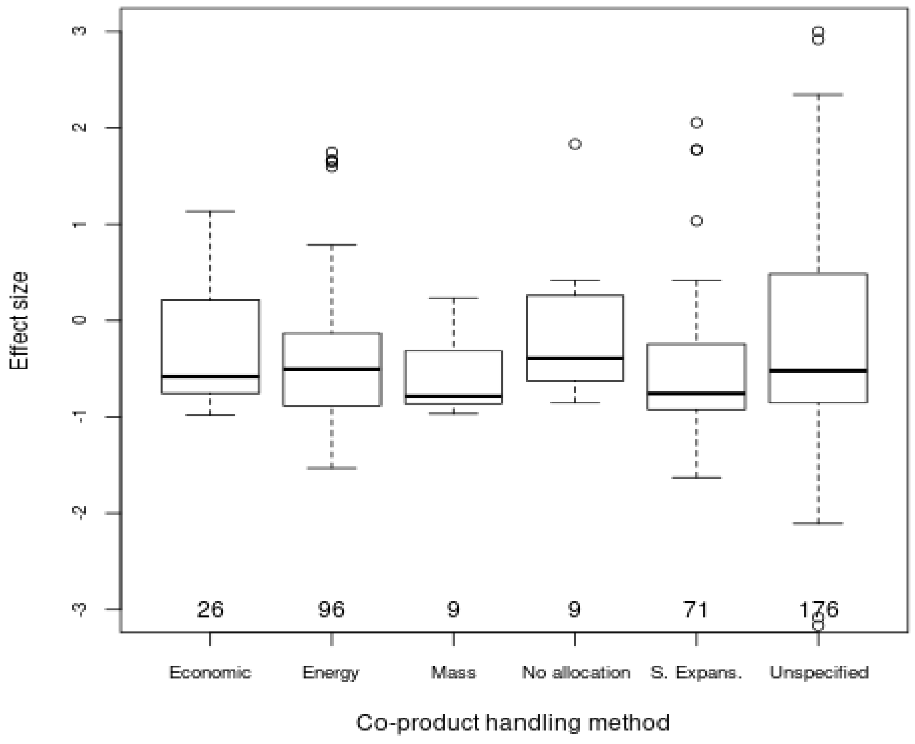

Table 1. Equation for the literature search on the Web of Science.

Number	Search equation
**#1**	TS=(life AND cycle) OR TS=lifecycle* OR TS=LCA OR TS=LCIA
**#2**	TS=balance OR TS=Analysis OR TS=impact* OR TS=accounting* OR TS=assessment* OR TS=quality OR TS=performance* OR TS=equity OR TS=externalit* OR TS=sustainability OR TS=valuation OR TS=evaluation
**#3**	TS=environment* AND #2
**#4**	#2 AND #1
**#5**	#3 OR #4
**#6**	TS=("greenhouse gas*" OR ghg OR biofuel*)
**#7**	#6 AND #2
**#8**	#7 OR #5
**#9**	TS="landuse change*" OR TS="land use change*" OR TS="landuse allocation*" OR TS="land use allocation*" OR TS="landuse dynamic*" OR TS="land use dynamic*" OR TS="land use option*" OR TS="landuse option*" OR TS="land use transition*" OR TS="landuse transition*" OR TS="land use conversion*" OR TS="landuse conversion*" OR TS="land use competition*" OR TS="landuse competition*" OR TS="land use take*" OR TS="landuse take*" OR TS="land use conversion*" OR TS="landuse conversion*" OR TS="land use scenari*" OR TS="Landuse scenari*" OR TS="land use strateg*" OR TS="Landuse strateg*" OR TS="land use impact*" OR TS="Landuse impact*" OR TS="land use competition*" OR TS="Landuse competition*" OR TS="land use expansion*" OR TS="Landuse expansion*" OR TS="land grabbing" OR TS="land sparring" OR TS="Land sharing" OR TS="agricultural expansion*" OR TS="Marginal land*" OR TS="land abandonment"
**#10**	#8 AND #9

List of references used in the meta-analysis

Acquaye, A. A., Sherwen, T., Genovese, A., Kuylenstierna, J., Koh, S. L., & McQueen-Mason, S. (2012). Biofuels and their potential to aid the UK towards achieving emissions reduction policy targets. *Renewable and Sustainable Energy Reviews*, *16*(7), 5414–5422.

Cherubini, F., & Ulgiati, S. (2010). Crop residues as raw materials for biorefinery systems – A LCA case study. *Applied Energy*, *87*(1), 47–57. https://doi.org/10.1016/j.apenergy.2009.08.024

Cocco, D., Deligios, P., Ledda, L., Sulas, L., Virdis, A., & Carboni, G. (2014). LCA Study of Oleaginous Bioenergy Chains in a Mediterranean Environment. *Energies*, *7*(10), 6258–6281. https://doi.org/10.3390/en7106258

De Souza, S. P., Pacca, S., de Ávila, M. T., & Borges, J. L. B. (2010). Greenhouse gas emissions and energy balance of palm oil biofuel. *Renewable Energy*, *35*(11), 2552–2561. https://doi.org/10.1016/j.renene.2010.03.028

Debolt, S., Campbell, J. E., SMITH Jr., R., Montross, M., & Stork, J. (2009). Life cycle assessment of native plants and marginal lands for bioenergy agriculture in Kentucky as a model for south-eastern USA. *GCB Bioenergy*, *1*(4), 308–316. https://doi.org/10.1111/j.1757-1707.2009.01023.x

Egeskog, A., Freitas, F., Berndes, G., Sparovek, G., & Wirsenius, S. (2014). Greenhouse gas balances and land use changes associated with the planned expansion (to 2020) of the sugarcane ethanol industry in Sao Paulo, Brazil. *Biomass and Bioenergy*, *63*, 280–290. https://doi.org/10.1016/j.biombioe.2014.01.030

Ericsson, N., Porsö, C., Ahlgren, S., Nordberg, Å., Sundberg, C., & Hansson, P.-A. (2013). Time-dependent climate impact of a bioenergy system - methodology development and application to Swedish conditions. *GCB Bioenergy*, *5*(5), 580–590. https://doi.org/10.1111/gcbb.12031

Falano, T., Jeswani, H. K., & Azapagic, A. (2014). Assessing the environmental sustainability of ethanol from integrated biorefineries. *Biotechnology Journal*, *9*(6), 753–765. https://doi.org/10.1002/biot.201300246

Gabrielle, B., Gagnaire, N., Massad, R. S., Dufossé, K., & Bessou, C. (2014). Environmental assessment of biofuel pathways in Ile de France based on ecosystem modeling. *Bioresource Technology*, *152*, 511–518. https://doi.org/10.1016/j.biortech.2013.10.104

García, C. A., Fuentes, A., Hennecke, A., Riegelhaupt, E., Manzini, F., & Masera, O. (2011). Life-cycle greenhouse gas emissions and energy balances of sugarcane ethanol production in Mexico. *Applied Energy*, *88*(6), 2088–2097. https://doi.org/10.1016/j.apenergy.2010.12.072

García, C. A., & Manzini, F. (2012). Environmental and economic feasibility of sugarcane ethanol for the Mexican transport sector. *Solar Energy*, *86*(4), 1063–1069. https://doi.org/10.1016/j.solener.2011.09.015

Gelfand, I ., Sahajpal, R ., Zhang, X ., Izaurralde, R. C., Gross, K. L., & Robertson, G.P. (2013). Sustainable bioenergy production from marginal lands in the US Midwest. Nature 493, 514–517.

Hamelin, L., Naroznova, I., & Wenzel, H. (2014). Environmental consequences of different carbon alternatives for increased manure-based biogas. *Applied Energy*, *114*, 774–782. https://doi.org/10.1016/j.apenergy.2013.09.033

Hansen, A., Meyer-Aurich, A., & Prochnow, A. (2013). Greenhouse gas mitigation potential of a second generation energy production system from short rotation poplar in Eastern Germany and its accompanied uncertainties. *Biomass and Bioenergy*, *56*, 104–115. https://doi.org/10.1016/j.biombioe.2013.05.004

Hassan, M. N. A., Jaramillo, P., & Griffin, W. M. (2011). Life cycle GHG emissions from Malaysian oil palm bioenergy development: The impact on transportation sector's energy security. *Energy Policy*, *39*(5), 2615–2625. https://doi.org/10.1016/j.enpol.2011.02.030

Iriarte, A., Rieradevall, J., & Gabarrell, X. (2012). Transition towards a more environmentally sustainable biodiesel in South America: The case of Chile. *Applied Energy*, *91*(1), 263–273. https://doi.org/10.1016/j.apenergy.2011.09.024

Iriarte, A., & Villalobos, P. (2013). Greenhouse gas emissions and energy balance of sunflower biodiesel: Identification of its key factors in the supply chain. *Resources, Conservation and Recycling*, *73*, 46–52. https://doi.org/10.1016/j.resconrec.2013.01.014

Kami Delivand, M., & Gnansounou, E. (2013). Life cycle environmental impacts of a prospective palm-based biorefinery in Pará State-Brazil. *Bioresource Technology*, *150*, 438–446. https://doi.org/10.1016/j.biortech.2013.07.100

Kauffman, N., Dumortier, J., Hayes, D. J., Brown, R. C., & Laird, D. A. (2014). Producing energy while sequestering carbon? The relationship between biochar and agricultural productivity. *Biomass and Bioenergy*, *63*, 167–176. https://doi.org/10.1016/j.biombioe.2014.01.049

Krohn, B. J., & Fripp, M. (2012). A life cycle assessment of biodiesel derived from the "niche filling" energy crop camelina in the USA. *Applied Energy*, *92*, 92–98. https://doi.org/10.1016/j.apenergy.2011.10.025

Lange, M. (2011). The GHG balance of biofuels taking into account land use change. Energy Policy 39, 2373–2385 .

Malça, J., & Freire, F. (2012). Addressing land use change and uncertainty in the life-cycle assessment of wheat-based bioethanol. *Energy*, *45*(1), 519–527. https://doi.org/10.1016/j.energy.2012.02.070

Meyer-Aurich, A., Schattauer, A., Hellebrand, H. J., Klauss, H., Plöchl, M., & Berg, W. (2012). Impact of uncertainties on greenhouse gas mitigation potential of biogas production from agricultural resources. *Renewable Energy*, *37*(1), 277–284. https://doi.org/10.1016/j.renene.2011.06.030

Mullins, K. A., Griffin, W. M., & Matthews, H. S. (2010). *Policy implications of uncertainty in modeled life-cycle greenhouse gas emissions of biofuels⊥*. ACS Publications. Retrieved from http://pubs.acs.org/doi/abs/10.1021/es1024993

Nasterlack, T., von Blottnitz, H., & Wynberg, R. (2014). Are biofuel concerns globally relevant? Prospects for a proposed pioneer bioethanol project in South Africa. *Energy for Sustainable Development*, *23*, 1–14. https://doi.org/10.1016/j.esd.2014.06.005

Nguyen, T. L. T., & Hermansen, J. E. (2012). System expansion for handling co-products in LCA of sugar cane bio-energy systems: GHG consequences of using molasses for ethanol production. *Applied Energy*, *89*(1), 254–261. https://doi.org/10.1016/j.apenergy.2011.07.023

Njakou Djomo, S., El Kasmioui, O., De Groote, T., Broeckx, L. S., Verlinden, M. S., Berhongaray, G., … Ceulemans, R. (2013). Energy and climate benefits of bioelectricity from low-input short rotation woody crops on agricultural land over a two-year rotation. *Applied Energy*, *111*, 862–870. https://doi.org/10.1016/j.apenergy.2013.05.017

Pradhan, A., D. S. Shrestha, D. S., J. Van Gerpen. J., McAloon, A., Yee,W., Haas,M., & Duffield, J. A. (2012). Reassessment of Life Cycle Greenhouse Gas Emissions for Soybean Biodiesel. *Transactions of the ASABE*, *55*(6), 2257–2264. https://doi.org/10.13031/2013.42483

Reinhard, J., & Zah, R. (2011). Consequential life cycle assessment of the environmental impacts of an increased rapemethylester (RME) production in Switzerland. *Biomass and Bioenergy*, *35*(6), 2361–2373. https://doi.org/10.1016/j.biombioe.2010.12.011

Saikkonen, L., Ollikainen, M., & Lankoski, J. (2014). Imported palm oil for biofuels in the EU: Profitability, greenhouse gas emissions and social welfare effects. *Biomass and Bioenergy*, *68*, 7–23. https://doi.org/10.1016/j.biombioe.2014.05.029

Searchinger, T., Heimlich, R., Houghton, R. A., Dong, F., Elobeid, A., Fabiosa, J., … Yu, T.-H. (2008). Use of U.S. Croplands for Biofuels Increases Greenhouse Gases Through Emissions from Land-Use Change. *Science*, *319*(5867), 1238–1240. https://doi.org/10.1126/science.1151861

Siangjaeo, S., Gheewala, S. H., Unnanon, K., & Chidthaisong, A. (2011). Implications of land use change on the life cycle greenhouse gas emissions from palm biodiesel production in Thailand. *Energy for Sustainable Development*, *15*(1), 1–7. https://doi.org/10.1016/j.esd.2011.01.002

Silalertruksa, T., & Gheewala, S. H. (2011a). Long-Term Bioethanol System and Its Implications on GHG Emissions: A Case Study of Thailand. *Environmental Science & Technology*, *45*(11), 4920–4928. https://doi.org/10.1021/es1040915

Silalertruksa, T., & Gheewala, S. H. (2011b). The environmental and socio-economic impacts of bio-ethanol production in Thailand. *Energy Procedia*, *9*, 35–43. https://doi.org/10.1016/j.egypro.2011.09.005

Silalertruksa, T., & Gheewala, S. H. (2012a). Environmental sustainability assessment of palm biodiesel production in Thailand. *Energy*, *43*(1), 306–314. https://doi.org/10.1016/j.energy.2012.04.025

Silalertruksa, T., & Gheewala, S. H. (2012b). Food, Fuel, and Climate Change: Is Palm-Based Biodiesel a Sustainable Option for Thailand? *Journal of Industrial Ecology*, *16*(4), 541–551. https://doi.org/10.1111/j.1530-9290.2012.00521.x

Silalertruksa, T., Gheewala, S. H., & Sagisaka, M. (2009). Impacts of Thai bio-ethanol policy target on land use and greenhouse gas emissions. *Applied Energy*, *86*, S170–S177. https://doi.org/10.1016/j.apenergy.2009.05.010

Smyth, B. M., & Murphy, J. D. (2011). The indirect effects of biofuels and what to do about them: the case of grass biomethane and its impact on livestock. *Biofuels, Bioproducts and Biorefining*, *5*(2), 165–184.

Souza, S. P., de Ávila, M. T., & Pacca, S. (2012). Life cycle assessment of sugarcane ethanol and palm oil biodiesel joint production. *Biomass and Bioenergy*, *44*, 70–79. https://doi.org/10.1016/j.biombioe.2012.04.018

Spatari, S., & MacLean, H. L. (2010). Characterizing Model Uncertainties in the Life Cycle of Lignocellulose-Based Ethanol Fuels. *Environmental Science & Technology*, *44*(22), 8773–8780. https://doi.org/10.1021/es102091a

Styles, D., & Jones, M. B. (2008). Miscanthus and willow heat production—An effective land-use strategy for greenhouse gas emission avoidance in Ireland? *Energy Policy*, *36*(1), 97–107. https://doi.org/10.1016/j.enpol.2007.08.030

Tonini, D., Hamelin, L., Wenzel, H., & Astrup, T. (2012). Bioenergy Production from Perennial Energy Crops: A Consequential LCA of 12 Bioenergy Scenarios including Land Use Changes. *Environmental Science & Technology*, *46*(24), 13521–13530. https://doi.org/10.1021/es3024435

Uusitalo, V., Havukainen, J., Kapustina, V., Soukka, R., & Horttanainen, M. (2014 a). Greenhouse Gas Emissions of Biomethane for Transport: Uncertainties and Allocation Methods. *Energy & Fuels*, *28*(3), 1901–1910. https://doi.org/10.1021/ef4021685

Uusitalo, V., Väisänen, S., Havukainen, J., Havukainen, M., Soukka, R., & Luoranen, M. (2014 b). Carbon footprint of renewable diesel from palm oil, jatropha oil and rapeseed oil. *Renewable Energy*, *69*, 103–113. https://doi.org/10.1016/j.renene.2014.03.020

Van Dam, J., Faaij, A. P. C., Hilbert, J., Petruzzi, H., & Turkenburg, W. C. (2009). Large-scale bioenergy production from soybeans and switchgrass in Argentina. *Renewable and Sustainable Energy Reviews*, *13*(8), 1679–1709. https://doi.org/10.1016/j.rser.2009.03.012

Van der Hilst, F., Verstegen, J. A., Zheliezna, T., Drozdova, O., & Faaij, A. P. (2014). Integrated spatiotemporal modelling of bioenergy production potentials, agricultural land use, and related GHG balances; demonstrated for Ukraine. *Biofuels, Bioproducts and Biorefining*, *8*(3), 391–411.

Wang, M., Han, J., Dunn, J. B., Cai, H., & Elgowainy, A. (2012). Well-to-wheels energy use and greenhouse gas emissions of ethanol from corn, sugarcane and cellulosic biomass for US use. *Environmental Research Letters*, *7*(4), 45905. https://doi.org/10.1088/1748-9326/7/4/045905

Wang, M. Q et al. (2011). Energy and greenhouse gas emission effects of corn and cellulosic ethanol with technology improvements and land use changes. Biomass and Bioenergy 35, 1885–1896

Wicke, B., Dornburg, V., Junginger, M., & Faaij, A. (2008). Different palm oil production systems for energy purposes and their greenhouse gas implications. *Biomass and Bioenergy*, *32*(12), 1322–1337. https://doi.org/10.1016/j.biombioe.2008.04.001

Yu, Y., & Wu, H. (2010). Bioslurry as a Fuel. 2. Life-Cycle Energy and Carbon Footprints of Bioslurry Fuels from Mallee Biomass in Western Australia. *Energy & Fuels*, *24*(10), 5660–5668. https://doi.org/10.1021/ef100957a

Data base

The data base used in the metaanalysis is available as a spreadsheet file.
